# Prevalence, predictors, and outcomes of major congenital anomalies: A population-based register study

**DOI:** 10.1038/s41598-023-27935-3

**Published:** 2023-02-07

**Authors:** Nader Al-Dewik, Muthanna Samara, Salma Younes, Rana Al-jurf, Gheyath Nasrallah, Sawsan Al-Obaidly, Husam Salama, Tawa Olukade, Sara Hammuda, Neil Marlow, Mohamed Ismail, Taghreed Abu Nada, M. Walid Qoronfleh, Binny Thomas, Ghassan Abdoh, Palli Valapila Abdulrouf, Thomas Farrell, Mai Al Qubaisi, Hilal Al Rifai

**Affiliations:** 1 Department of Research, Women’s Wellness and Research Center, Hamad Medical Corporation, P.O. Box 3050, Doha, Qatar; 2 Translational Research Institute (TRI), Hamad Medical Corporation (HMC), Doha, Qatar; 3 Genomics and Precision Medicine (GPM), College of Health and Life Science (CHLS), Hamad Bin Khalifa University (HBKU), 34110 Doha, Qatar; 4 Faculty of Health and Social Care Sciences, Kingston University, St. George’s University of London, London, UK; 5 Department of Pediatrics and Neonatology, Neonatal Intensive Care Unit, Newborn Screening Unit, Women’s Wellness and Research Center, Hamad Medical Corporation, Doha, Qatar; 6 Department of Psychology, Kingston University London, Kingston upon Thames, UK; 7 Department of Biomedical Science, College of Health Sciences, Member of QU Health, Qatar University, Doha, Qatar; 8 Obstetrics and Gynecology Department, Women’s Wellness and Research Center, Hamad Medical Corporation, Doha, Qatar; 9 Institute for Women’s Health, UCL, London, UK; 10 Q3CG Research Institute, Research & Policy Division, 7227 Rachel Drive, Ypsilanti, MI 48917 USA; 11 Department of Pharmacy, Women’s Wellness and Research Center, Hamad Medical Corporation, Doha, Qatar

**Keywords:** Risk factors, Congenital heart defects

## Abstract

Congenital anomalies (CAs) are a leading cause of morbidity and mortality in early life. We aimed to assess the incidence, risk factors, and outcomes of major CAs in the State of Qatar. A population-based retrospective data analysis of registry data retrieved from the Perinatal Neonatal Outcomes Research Study in the Arabian Gulf (PEARL-Peristat Study) between April 2017 and March 2018. The sample included 25,204 newborn records, which were audited between April 2017 and March 2018, of which 25,073 live births were identified and included in the study. Maternal risk factors and neonatal outcomes were assessed for association with specific CAs, including chromosomal/genetic, central nervous system (CNS), cardiovascular system (CVS), facial, renal, multiple congenital anomalies (MCAs) using univariate and multivariate analyses. The incidence of any CA among live births was 1.3% (n = 332). The most common CAs were CVS (n = 117; 35%), MCAs (n = 69, 21%), chromosomal/genetic (51; 15%), renal (n = 39; 12%), CNS (n = 20; 6%), facial (14, 4%), and other (GIT, Resp, Urogenital, Skeletal) (n = 22, 7%) anomalies. Multivariable regression analysis showed that multiple pregnancies, parity ≥ 1, maternal BMI, and demographic factors (mother’s age and ethnicity, and infant’s gender) were associated with various specific CAs. In-hospital mortality rate due to CAs was estimated to be 15.4%. CAs were significantly associated with high rates of caesarean deliveries (aOR 1.51; 95% CI 1.04–2.19), Apgar < 7 at 1 min (aOR 5.44; 95% CI 3.10–9.55), Apgar < 7 at 5 min (aOR 17.26; 95% CI 6.31–47.18), in-hospital mortality (aOR 76.16; 37.96–152.8), admission to neonatal intensive care unit (NICU) or perinatal death of neonate in labor room (LR)/operation theatre (OT) (aOR 34.03; 95% CI 20.51–56.46), prematurity (aOR 4.17; 95% CI 2.75–6.32), and low birth weight (aOR 5.88; 95% CI 3.92–8.82) before and after adjustment for the significant risk factors. This is the first study to assess the incidence, maternal risk factors, and neonatal outcomes associated with CAs in the state of Qatar. Therefore, a specialized congenital anomaly data registry is needed to identify risk factors and outcomes. In addition, counselling of mothers and their families may help to identify specific needs for pregnant women and their babies.

## Introduction

A congenital anomaly (CA), or birth defects is a structural, functional, behavioral, or metabolic abnormality present during intrauterine life which may be identified prenatally, at birth, or detected later in infancy^1,2^. They add a great burden to global public health as they cause early miscarriage, fetal death, infant death. CAs contribute to childhood disabilities significantly impacting individuals and families, health-care systems and societies^2,3^.

CAs are classified according to the International Classification of Diseases by the affected body system^4^, where major anomalies affect the infant’s life expectancy, health status, physical or social functioning. On other hand, “minor” anomalies are those with little or no impact on health or short-term or long-term function^4^. The March of Dimes Global Report on Birth Defects estimates that 8 million infants are born each year world-wide with major CAs, comprising approximately 6% of newborns^2,5^, contributing to 300,000 annual deaths in the first four weeks after birth^2,5^. Birth prevalence of CAs has been shown to vary greatly from country to country. According to the report, among the 193 reporting countries the world’s highest rates of birth defects are in the Middle East and North Africa^5^, and range from 82 per 1000 live births in Sudan to 39.7 per 1000 in France^5^. The prevalence of CAs in low-income, developing, and developed countries is 64.2, 55.7, and 47.2 per 1000 live births respectively^5^. The variation in rates may be explained by social, racial, ecological, and economical influences^6,7^.

Qatar ranks 16th globally in this report^5^. CAs are the leading cause of infant mortality in the country, accounting for 34.5% of total infant mortality^8^. CAs are estimated to comprise 2.13% of total deaths, with an age-adjusted death rate of approximately 4.95 per 100,000 of population^9^. In 2018, neonatal and infant mortality rates in Qatar were 3.5 and 6.21 deaths per 1000 live births, respectively^10^.

Risk factors for CAs are defined as predisposing factors that may contribute singly or interactively to induce structural or functional abnormalities of neonates^11^ and may be attributed to genetics, exposure to chemical, physical and biological hazards, or other maternal elements^11^. They are often associated with adverse perinatal outcomes, including preterm birth, intrauterine growth restriction and stillbirth^12^. Epidemiological studies have identified maternal age over 35 years, parity, mode of pregnancy, pregnancy type (singleton or multiple), and concurrent maternal disease as the main contributing risk factors for birth defects^13–19^.

Due to serious potential impact on health, wellness and survival, the World Health Assembly emphasized CAs as global public health priority in 2010 and addressed the urgent need for action^20^. Early identification of maternal and neonatal risk factors and accurate quantification of CAs within a given population is crucial for estimating their burden, documenting the need for prevention, for enabling public health policy development, as well as the planning, evaluation and implementation of preventive measures and treatment services^12^. Given the fact that Qatar is among the top 20 countries with the highest rates of birth defects, and that up to 70% of the cases can either be prevented, or offered care that could be lifesaving^5^, it is crucial to estimate the burden of disease, identify risk factors, and assess the outcomes. In this study we assess the prevalence and distribution of CAs in Qatar, with a specific focus on the associated maternal risk factors, and pregnancy and neonatal outcomes.

## Methods

### Study population

The study population included live births at 24^+0^ weeks of gestation and above whose mothers delivered between April 2017 and March 2018 at Hamad Medical Corporation (HMC). HMC is the main provider of secondary and tertiary healthcare in Qatar. HMC is also one of the leading hospital providers in the Middle East. HMC comprises the national hospital and the main multiple regional hospitals that are widely distributed in Qatar. These hospitals account for the majority of births in the country. Stillbirths were excluded, as there was no certainty of the presence or absence of congenital anomalies. This study complies with the World Medical Association Declaration of Helsinki regarding the ethical conduct of research and was approved by Hamad Medical Corporation Institutional Review Board, with a waiver of consent.

### Data collection

This was a 12-month retrospective population-based study conducted using registry data from the Perinatal Neonatal Outcomes Research Study in the Arabian Gulf (PEARL-Peristat Study), Qatar^21–23^. The PEARL-Peristat Study is an ongoing cohort study based on the predesigned hospital data pertaining to mothers and their newborns^21–23^. We followed the WHO international classification of diseases to classify CAs according to the affected body system (see Appendix 1)^4^. We only included major CAs that affect the infant’s life expectancy, health status, physical or social functioning. Data were captured and retrieved from the PEARL-Peristat Study using the International Classification of Diseases Clinical Modification Codes, 10^th^ revision (ICD-10). ICD codes were assigned by trained coders. ICD-10 codes were later used as a guidance and retrieval index of the collected CAs.

We used seven categories for classification of anomalies: chromosomal/genetic, central nervous system (CNS), cardiovascular system (CVS), facial, renal, multiple CAs (MCAs), and other CAs (gastrointestinal; GIT, respiratory, urogenital, and skeletal). Maternal factors included age of the mother at conception (20–34 years vs. < 20 years and ≥ 35 years), nationality (Qataris and other Arabs vs. other nationalities), consanguinity (yes; the mother and the father are related to each other in any level of relatedness vs. no)^22–24^, parity (nulliparous vs. parity ≥ 1), maternal BMI (normal vs. underweight, overweight, and obese), pregnancy mode (spontaneous vs. assisted conception), pregnancy type (singleton vs. multiple), chronic hypertension (yes vs. no), delivery mode (vaginal vs. caesarean), and history of maternal illness such as diabetes. Diabetes included three categories: none, gestational diabetes mellitus (GDM), and pre-gestational diabetes mellitus (PGDM) including type 1 and type 2 diabetes. Neonatal factors included data about gestational age at time of delivery (Full term: ≥  37 weeks of gestational age vs. preterm: ≤ 36 weeks of gestational age), birth weight (≤ 2499 g vs. ≥ 2500 g), the immediate birth status (Apgar score < 7 at 1 and at 5 min), macrosomic baby (< 4 kg vs. ≥ 4 kg) and infant gender (male vs. female). Newborn outcomes included two factors; the first factor, before discharge criterion, which includes admission to routine postnatal ward versus admission to NICU or perinatal death in the labour room/operation theatre, and the second factor included discharged babies versus in-hospital death.

### Statistical analysis

Statistical analysis was performed using IBM SPSS 28 software (SPSS Chicago IL, USA). Descriptive statistics were reported for the characteristics of maternal and neonatal groups. Newborns with multiple anomalies were counted for each specific form of congenital abnormality. The overall prevalence of CAs and their subtypes by maternal, neonatal and baby outcome factors were analyzed using Chi Square analysis.

We then performed a series of logistic regression analyses to investigate the risk factors and consequences of CAs. Firstly, logistic regression analysis was performed for the above maternal or perinatal risk factors to examine their associations with CAs using univariate logistic regression. Subsequent multiple logistic regression was performed using variables which were significantly associated with CAs in the univariate analysis to investigate their independent association.

Secondly, univariate logistic regression was performed to investigate the association between CAs and number of outcomes (delivery mode, Apgar < 7 at 1 min, Apgar < 7 at 5 min, admission to NICU/perinatal death in LR/OT, in-hospital mortality, prematurity, macrosomia, and low birthweight). Multiple logistic regression was then performed adjusting for all significant variables of CAs from the univariate analysis from the first stage. Crude Odds Ratios (Ors) (from the univariate analysis) and adjusted OR (from the multiple logistic regression analysis) and 95% Confidence Interval (CI) were reported. The statistical significance was set at p < 0.05.

### Ethics approval and consent to participate

The study was approved by the Hamad Medical Corporation Institutional Review Board, with a waiver of consent.

## Results

### Characteristics of the study population

**Figure 1:**
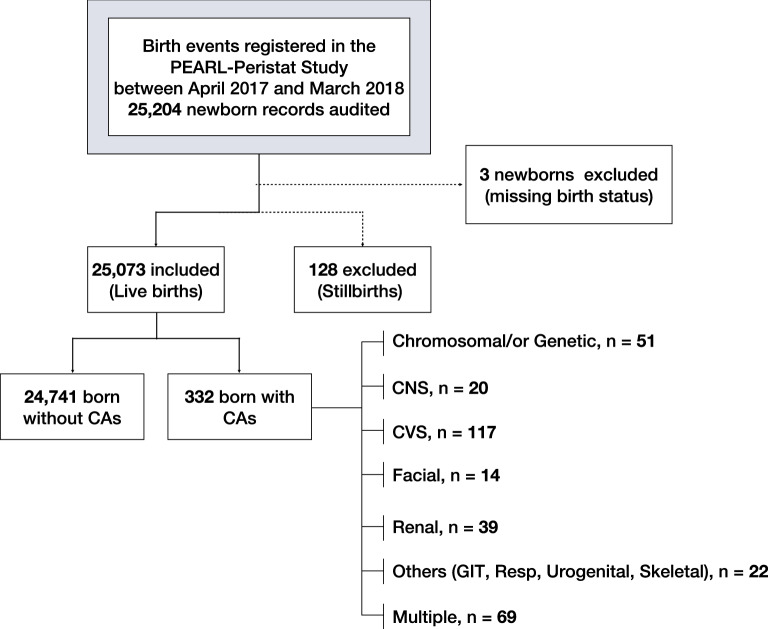
Flow diagram of study sample size of the population-based retrospective of registry data retrieved from the PEARL-Peristat study between April 2017 and March 2018.

Of the birth events to 24,660 mothers registered in the PEARL-Peristat Study, 25,204 newborn records were included, of which 25,073 were live births and 128 were stillbirths; birth status was missing for 3 babies. CAs were recognized in 332 infants (1.3%) (Fig. 1).

Mothers who gave birth to infants with CAs had similar distributions of nationality, maternal age, parity, chronic hypertension, consanguinity, microsomic baby, gender, and maternal BMI to those who did not. On the other hand, mothers who gave birth to infants with CAs had more frequently assisted conception, multiple pregnancies or had mothers with diabetes. Infants with CAs were more frequently born by caesarean section, more frequently preterm and low birth weight, and were in poorer condition at birth, as recorded by the Apgar score and less likely to be admitted to the postnatal ward or survive overall (Table 1).

**Table 1:** Neonatal & maternal socio-demographic factors of study population (N; %, the dominator number is indicated when there is missing data).

**Risk factors and outcomes**	**Any congenital anomaly**			P-value
	Yes (n = 332) n (%)	No (n = 24,741) n (%)	Total (n = 25,073) n (%)	
**Nationality**^†^				0.102
Qatari	93 (28)	5770/24,737 (23.3)	5863/25,069 (23.4)	
Other arabs	138 (41.6)	10,483/24,737 (42.4)	10,621/25,069 (42.4)	
Other nationalities	101 (30.4)	8484/24,737 (34.3)	8585/25,069 (34.2)	
**Maternal age**				0.185
20–34 years	250 (75.3)	19,579 (79.1)	19,829 (79.1)	
< 20 years	6 (1.8)	482 (1.9)	488 (1.9)	
≥ 35 years	76 (22.9)	4680 (18.9)	4756 (19)	
**Pregnancy type**				**0.000**
Singleton	297 (89.5)	23,739 (96)	24,036 (95.9)	
Multiple	35 (10.5)	1002 (4)	1037 (4.1)	
**Parity**^†^				0.491
Nulliparous	100 (30.1)	7026/24,737 (28.4)	7126/25,069 (28.4)	
Parity ≥ 1	232 (69.9)	17,711/24,737 (71.6)	17,943/25,069 (71.6)	
**Diabetes**				**0.017**
None	234 (70.5)	17,170 (69.4)	17,404/18,693 (69.4)	
GDM	83 (25)	7000 (28.3)	7083/18,693 (28.2)	
PGDM	15 (4.5)	571 (2.3)	586/18,693 (2.3)	
**Chronic hypertension**				0.978
No	328 (98.8)	24,447 (98.8)	24,775 (98.8)	
Yes	4 (1.2)	294 (1.2)	298 (1.2)	
**Consanguinity**^†^				0.115
No	65/104 (62.5)	5832/8372 (69.7)	5897/8476 (69.6)	
Yes	39/104 (37.5)	2540/8372 (30.3)	2579/8476 (30.4)	
**Pregnancy mode**^†^				**0.000**
Spontaneous	299/330 (90.6)	23,688/24,603 (96.3)	23,987/24,933 (96.2)	
Assisted conception	31/330 (9.4)	915/24,603 (3.7)	946/24,933 (3.8)	
**Delivery mode**				**0.000**
Vaginal	170 (51.2)	16,347 (66.1)	16,517 (65.9)	
Caesarean	162 (48.8)	8394 (33.9)	8556 (34.1)	
**Gestational age at birth**				**0.000**
24–31 weeks	43 (13)	376 (1.5)	419 (1.7)	
32–36 weeks	73 (22)	2028 (8.2)	2101 (8.4)	
≥ 37 weeks	216 (65.1)	22,337 (90.3)	22,553 (89.9)	
**Birth weight**^†^				**0.000**
≤ 2499 g	124/331 (37.5)	2276/24,733 (9.2)	2400/25,064 (9.6)	
≥ 2500 g	207/331 (62.5)	22,457/24,733 (90.8)	22,664/25,064 (90.4)	
**Macrosomic baby**^†^				0.073
< 4 kg	322/331 (97.3)	23,536/24,733 (95.2)	23,858/25,064 (95.2)	
≥ 4 kg	9/331 (2.7)	1197/24,733 (4.8)	1206/25,064 (4.8)	
**Apgar < 7 at 1 min**				**0.000**
No	270 (81.3)	24,168 (97.7)	24,438 (97.5)	
Yes	62 (18.7)	573 (2.3)	635 (2.5)	
**Apgar < 7 at 5 min**				**0.000**
No	309 (93.1)	24,686 (99.8)	24,995 (99.7)	
Yes	23 (6.9)	55 (0.2)	78 (0.3)	
**Gender**^†^				0.294
Male	179 (53.9)	12,619/24,735 (51)	12,798/25,067 (51.1)	
Female	153 (46.1)	12,116/24,735 (49)	12,269/25,067 (48.9)	
**Baby outcome**^†^				**0.000**
Discharged alive	281 (84.6)	24,693/24,739 (99.8)	24,974/25,071 (99.6)	
Died in hospital	51 (15.4)	46/24,739 (0.2)	97/25,071 (0.4)	
**Baby disposition**^†^				**0.000**
Postnatal ward	46 (13.9)	21,056 (85.1)	21,102 (84.2)	
NICU or died in LR/OT	286 (86.1)	3682 (14.9)	3968 (15.8)	
**Maternal BMI**				0.164
Normal	37/128 (28.9)	3282/9606 (34.2)	3319/9734 (34.1)	
Underweight	7/128 (5.5)	250/9606 (2.6)	257/9734 (2.6)	
Overweight	43/128 (33.6)	3171/9606 (33)	3214/9734 (33)	
Obese	41/128 (32)	2903/9606 (30.2)	2944/9734 (30.2)	

*GDM* gestational diabetes mellitus, *PGDM* pregestational diabetes mellitus.

^†^Missing data for: nationality (n = 4), parity (n = 4), consanguinity (n = 16,597), maternal BMI (15,339), pregnancy mode (n = 140), birth weight/macrosomia (n = 9), gender (n = 4), baby outcome (n = 2).

Significant values are in bold.

The prevalence of congenital anomalies was as follows: CVS (n = 117; 35%), multiple system anomalies (n = 69; 21%), chromosomal/genetic (n = 51; 15%), renal (n = 39; 12%), CNS (n = 20; 6%), facial (n = 14; 4%), and other (GIT, Resp, Urogenital, Skeletal) (n = 22, 7%) anomalies.

The maternal, medical and newborn characteristics distribution of the overall study population classified by each CA type are summarized in Appendix 2.

### Risk factors for congenital anomalies

#### Any congenital anomaly

Univariate logistic regression analysis revealed significant associations for Qataris, multiple pregnancy, PGDM, assisted conception, and maternal BMI (underweight women vs. normal) with any CA (Table 2). No significant associations were observed for maternal age, parity, GDM, chronic hypertension, consanguinity, macrosomia, or gender. In the multivariate analysis, when entering all significant variables from the univariate regression analysis together, no significant associations remained (Table 2).

**Table 2:** Risk factors associated with the occurrence of congenital anomalies including univariate regression analysis (crude odds ratio: cOR) and multiple regression analysis (adjusted odds ratio: aOR) adjusting for the significant factors from the univariate analysis.

Variables	**Any CA (n = 332)**		**Chromosomal/genetics (n = 51)**		**CNS (n = 20)**		**CVS (n = 117)**		**Facial (n = 14)**		**Renal (n = 39)**		**Others congenital anomalies (n = 22)**		**Multiple congenital anomalies (n = 69)**	
	**c OR (95% CI)**	**a OR (95% CI)**	**c OR (95% CI)**	**a OR (95% CI)**	**c OR (95% CI)**	**a OR (95% CI)**	**c OR (95% CI)**	**a OR (95% CI)**	**c OR (95% CI)**	**a OR (95% CI)**	**c OR (95% CI)**	**a OR (95% CI)**	**c OR (95% CI)**	**a OR (95% CI)**	**c OR (95% CI)**	**a OR (95% CI)**
**Nationality**																
Qatari	1.35 (1.02–1.8)^‡^	1.44 (0.89–2.33)	0.77 (0.37–1.6)		0.88 (0.21–3.69)		1.32 (0.83–2.09)		1.47 (0.3–7.29)		2.57 (1.08–6.14)^‡^	7.3 (1.45–36.86)^‡^	3.68 (0.71–18.95)	2.99 (0.57–15.6)	1.41 (0.79–2.5)	
Other arabs	1.11 (0.85–1.43)	1.08 (0.7–1.66)	0.73 (0.39–1.36)		1.94 (0.68–5.52)		0.96 (0.62–1.48)		2.16 (0.57–8.14)		1.72 (0.74–3.99)	2.85 (0.57–14.21)	6.07 (1.39–26.55)^‡^	5.5 (1.25–24.13)^‡^	0.74 (0.42–1.32)	
Other nationalities	Ref		Ref		Ref		Ref		Ref		Ref		Ref		Ref	
**Maternal age**																
20–34 years	Ref		Ref		Ref		Ref		Ref		Ref		Ref		Ref	
< 20 years	0.97 (0.43–2.2)		0 (0–)	0 (0–)	0 (0–)		0.45 (0.06–3.21)		3.39 (0.44–26.08)		1.35 (0.18–9.95)		0 (0–)		2.34 (0.73–7.53)	
≥ 35 years	1.27 (0.98–1.65)		3.17 (1.82–5.53)*	3.4 (1.37–8.44)^†^	0.46 (0.11–2)		1.15 (0.74–1.79)		0.35 (0.05–2.68)		1.12 (0.51–2.44)		0.93 (0.31–2.75)		1.13 (0.62–2.03)	
**Pregnancy type**																
Singleton	Ref		Ref		Ref		Ref		Ref		Ref		Ref		Ref	
Multiple	2.79 (1.96–3.99)*	1.32 (0.64–2.72)	0 (0–)		5.92 (1.98–17.75)^†^	5.92 (1.98–17.75)^†^	3.48 (2.02–6.01)*	2.93 (1.5–5.74)^†^	1.82 (0.24–13.94)		2.71 (0.96–7.63)		6.97 (2.57–18.92)*	4.25 (1.12–16.15)^‡^	2.26 (0.97–5.23)	
**Parity**																
Nulliparous	Ref		Ref		Ref		Ref		Ref		Ref		Ref		Ref	
Parity ≥ 1	0.92 (0.73–1.17)		2.13 (1–4.54)^‡^	3,721,812.484 (0–)	0.74 (0.29–1.85)		1.15 (0.76–1.74)		0.99 (0.31–3.16)		0.46 (0.25–0.87)^‡^	0.21 (0.06–0.68)^†^	1.79 (0.6–5.28)		0.55 (0.34–0.88)^‡^	0.84 (0.33–2.130.72)
**Diabetes**																
None	Ref		Ref		Ref		Ref		Ref		Ref		Ref		Ref	
GDM	0.87 (0.68–1.12)	1.01 (0.69–1.49)	1.15 (0.64–2.09)		1.05 (0.4–2.74)		1.02 (0.68–1.54)		0.67 (0.19–2.4)		0.51 (0.21–1.22)	1.26 (0.38–4.14)	0.92 (0.36–2.35)		0.63 (0.34–1.15)	0.41 (0.14–1.240.12)
PGDM	1.93 (1.14–3.27)^‡^	1.75 (0.83–3.71)	0.88 (0.12–6.47)		0 (0–)		1.9 (0.77–4.72)		0 (0–)		4.15 (1.45–11.84)^†^	0 (0–)	0 (0–)		2.95 (1.17–7.41)^‡^	2.55 (0.72–9.050.15)
**Chronic hypertension**																
No	Ref		Ref		Ref		Ref		Ref		Ref		Ref		Ref	
Yes	1.01 (0.38–2.74)		1.66 (0.23–12.08)		0 (0–)		0.72 (0.1–5.15)		0 (0–)		2.19 (0.3–15.99)		0 (0–)		1.22 (0.17–8.84)	
**Consanguinity**																
No	Ref		Ref		Ref		Ref		Ref		Ref		Ref		Ref	
Yes	1.38 (0.92–2.05)		1.46 (0.57–3.77)		1.53 (0.26–9.17)		1.15 (0.57–2.3)		1,908,041.2 (0–)		1.53 (0.54–4.3)		1.53 (0.26–9.17)		1.07 (0.44–2.63)	
**Pregnancy mode**																
Spontaneous	Ref		Ref		Ref		Ref		Ref		Ref		Ref		Ref	
Assisted conception	2.68 (1.84–3.91)*	1.88 (0.95–3.72)	1.06 (0.26–4.35)		2.88 (0.67–12.42)		2.71 (1.45–5.07)^†^	1.46 (0.68–3.16)	4.31 (0.96–19.31)		1.4 (0.34–5.81)		5.75 (1.94–17.03)^†^	2.26 (0.53–9.68)	3.45 (1.65–7.24)^†^	3.48 (1.15–10.570.03)^‡^
**Gender**																
Male	1.12 (0.9–1.4)		0.92 (0.53–1.6)		0.52 (0.21–1.3)		1.16 (0.8–1.67)		1.73 (0.58–5.16)		1.24 (0.66–2.34)		3.26 (1.2–8.85)^‡^	3.29 (1.21–8.93)^‡^	0.99 (0.62–1.59)	
Female	Ref		Ref		Ref		Ref		Ref		Ref		Ref		Ref	
**Maternal BMI**																
Normal	Ref		Ref		Ref		Ref		Ref		Ref		Ref		Ref	
Underweight	2.48 (1.1–5.63)^‡^	2.21 (0.92–5.3)	5.25 (1.01–27.2)^‡^	7.6 (1.44–40.01)^‡^	13.13 (0.82–210.51)		1.01 (0.13–7.75)		0.000002 (0–)		1.31 (0.17–10.3)	1.21 (0.15–9.63)	0 (0–)		6.56 (1.2–36.01)^‡^	3.18 (0.35–28.730.30)
Overweight	1.2 (0.77–1.87)	1.15 (0.74–1.8)	1.45 (0.46–4.57)	1.01 (0.32–3.22)	1.04 (0.06–16.55)		1.59 (0.79–3.21)		4 (0.5–40)		0.21 (0.05–0.95)^‡^	0.23 (0.05–1.08)	1.04 (0.21–5.13)		1.55 (0.44–5.51)	1.63 (0.45–5.840.45)
Obese	1.25 (0.8–1.96)	1.11 (0.7–1.76)	1.36 (0.41–4.45)	0.75 (0.22–2.52)	1.13 (0.07–18.08)		1.57 (0.77–3.2)		0.000002 (0–)		0.11 (0.01–0.88)^‡^	0.13 (0.02–1.08)	1.13 (0.23–5.61)		3.39 (1.09–10.53)^‡^	3.43 (1.06–11.120.04)^‡^

*CNS* central nervous system; *CVS* cardiovascular system; others: gastrointestinal, respiratory, urogenital, and skeletal; multiple: two or more anomalies; *GDM* gestational diabetes mellitus; *PGDM* pregestational diabetes mellitus.

*p < 0.001; ^†^p < 0.01; ^‡^p < 0.05.

#### Chromosomal/genetic anomalies

Univariate logistic regression analysis revealed significant associations for maternal age (≥ 35 years), parity ≥ 1, and maternal BMI (underweight women vs. normal) with chromosomal/genetic anomalies (Table 2). No significant associations were observed for nationality, age < 20 years, pregnancy type, diabetes status, chronic hypertension, consanguinity, pregnancy mode, macrosomia, or baby gender. In the multivariate analysis, when entering all significant factors from the univariate regression analysis, significant associations were observed for maternal age ≥ 35 years and maternal BMI (underweight women) only (Table 2).

#### Central nervous system (CNS) anomalies

Univariate logistic regression analysis showed significant associations for multiple pregnancy with CNS abnormalities (Table 2). No significant associations were observed for nationality, maternal age, parity, GDM, PGDM, chronic hypertension, consanguinity, pregnancy mode, baby gender, and maternal BMI (Table 2). In the multivariate analysis, when entering all significant factors from the univariate regression analysis, significant association was still being observed for multiple pregnancies (Table 2).

#### Cardiovascular system (CVS) anomalies

Univariate logistic regression analysis revealed significant associations for multiple pregnancy and assisted conception with CVS anomalies (Table 2). No significant associations were observed for nationality, maternal age, parity, GDM, PGDM, chronic hypertension, consanguinity, baby gender, and maternal BMI (Table 2). In the multivariate analysis, when entering all significant factors from the univariate regression analysis, significant association was observed for multiple pregnancies only.

#### Congenital facial anomalies

Univariate logistic regression analysis revealed no significant associations with congenital facial abnormalities for any of the risk factors (Table 2).

#### Congenital renal anomalies

Univariate logistic regression analysis revealed that nationality (Qatari), Parity ≥ 1, PGDM, and maternal BMI (less likely to be overweight and obese in comparison to normal weight) are significantly more likely to be associated with congenital renal abnormalities (Table 2). No significant associations were observed for other Arab nationalities, maternal age, multiple pregnancies, GDM, chronic hypertension, consanguinity, assisted conception, gender or underweight women (Table 2). In the multivariate analysis, when entering all significant factors from the univariate regression analysis, significant associations were observed for infants born to Qatari mothers and parity ≥ 1 only (Table 2).

#### Other congenital anomalies (GIT, respiratory, urogenital, and skeletal anomalies)

Univariate logistic regression analysis revealed that nationality (Non-Qatari Arabs), multiple pregnancies, assisted conception, and male babies are significantly associated with other CAs (GIT, respiratory, urogenital, and skeletal anomalies) (Table 2). No significant associations were observed for Qataris, maternal age, Parity ≥ 1, GDM, PGDM, consanguinity, chronic hypertension, and maternal BMI (Table 2). In the multivariate analysis, when entering all significant factors from the univariate regression analysis, significant associations were observed for infants born to Non-Qatari Arab mothers, multiple pregnancies, and male infants only (Table 2).

#### Multiple congenital anomalies (MCAs)

Univariate logistic regression analysis revealed that Parity ≥ 1, PGDM, assisted conception, and maternal BMI (underweight and obese women vs. normal) are significantly associated with MCAs (Table 2). No significant associations were observed for nationality, maternal age, multiple pregnancies, GDM, chronic hypertension, consanguinity, baby gender, and overweight mothers (Table 2). In the multivariate analysis, when entering all significant factors from the univariate regression analysis, significant associations were observed for assisted conception, and maternal BMI (obese women) only (Table 2).

### Consequences of congenital anomalies

#### Any congenital anomaly

Any CAs were significantly associated with various outcomes including higher rates of caesarean deliveries, Apgar < 7 at 1 min and at 5 min, in hospital mortality, admission to NICU or perinatal death in LR/OT, prematurity and low birth weight before and after adjustment for nationality, pregnancy type, diabetes, pregnancy mode, and maternal BMI (Table 3).

**Table 3:** Pregnancy outcomes of the overall study population according to the presence of congenital anomalies.

	**Caesarean delivery**		**Apgar < 7 at 1 min**		**Apgar < 7 at 5 min**		**In-hospital mortality**		**Admission to NICU or perinatal death in LR/OT**		**Macrosomia**		**Prematurity**		**Low birthweight**	
	**cOR (95%CI)**	**aOR (95%CI)**	**cOR (95%CI)**	**aOR (95%CI)**	**cOR (95%CI)**	**aOR (95%CI)**	**cOR (95%CI)**	**aOR (95%CI)**	**cOR (95%CI)**	**aOR (95%CI)**	**cOR (95%CI)**	**aOR (95%CI)**	**cOR (95%CI)**	**aOR (95%CI)**	**cOR (95%CI)**	**aOR (95%CI)**
**Any congenital anomalies**	1.86 (1.49–2.31)*	1.51 (1.04–2.19)^‡^	9.69 (7.26–12.92)*	5.44 (3.1–9.55)*	33.41 (20.27–55.05)*	17.26 (6.31–47.18)*	97.43 (64.3–147.62)*	76.16 (37.96–152.8)*	35.55 (25.99–48.64)*	34.03 (20.51–56.46)*	0.55 (0.28–1.07)	0.72 (0.26–1.96)	4.99 (3.97–6.28)*	4.17 (2.75–6.32)*	5.91 (4.71–7.41)*	5.88 (3.92–8.82)*
Nationality																
Qatari		0.88 (0.78–1)^‡^		1.45 (1.02–2.07)^‡^		1.53 (0.51–4.61)		2.16 (0.86–5.43)		0.95 (0.81–1.12)		0.83 (0.61–1.13)		1.25 (1.03–1.52)^‡^		1.61 (1.32–1.96)*
Other arabs		0.91 (0.82–1)		0.96 (0.69–1.34)		1.27 (0.47–3.43)		1 (0.4–2.5)		0.9 (0.79–1.03)		1.54 (1.23–1.92)*		0.92 (0.77–1.1)		0.75 (0.63–0.91)^†^
Other nationalities		Ref		Ref		Ref		Ref		Ref		Ref		Ref		Ref
Pregnancy type																
Singleton		Ref		Ref		Ref		(–)		Ref		Ref		Ref		Ref
Multiple		7.19 (5.61–9.21)*		2.6 (1.61–4.2)*		2.57 (0.69–9.53)		2.41 (0.72–8.09)		5.4 (4.34–6.71)*		0.05 (0.01–0.38)^†^		20.3 (16.09–25.6)*		24.83 (19.57–31.49)*
Diabetes																
None		Ref		Ref		Ref		(–)		Ref		Ref		Ref		Ref
GDM		1.02 (0.93–1.12)		0.98 (0.73–1.32)		0.92 (0.38–2.18)		1.27 (0.61–2.64)		1.12 (0.99–1.27)		1.04 (0.85–1.27)		1.12 (0.95–1.31)		0.95 (0.8–1.13)
PGDM		2.92 (2.31–3.68)*		1.97 (1.16–3.35)^‡^		0.86 (0.11–6.65)		0 (0–)		2.49 (1.93–3.2)*		1.28 (0.8–2.05)		2.86 (2.13–3.83)*		1.29 (0.89–1.86)
Pregnancy mode																
Spontaneous		Ref		Ref		Ref		(–)		Ref		Ref		Ref		Ref
Assisted conception		1.6 (1.28–2)*		0.91 (0.52–1.6)		1.07 (0.24–4.71)		0.73 (0.19–2.76)		1.42 (1.12–1.81)^†^		0.37 (0.15–0.91)^‡^		1.67 (1.27–2.2)*		1.62 (1.22–2.14)^†^
Maternal BMI																
Normal																
Underweight		0.56 (0.4–0.79)^†^		0.68 (0.25–1.88)		0 (0–)		0.97 (0.11–8.25)		1.09 (0.76–1.56)		0.59 (0.24–1.45)		1.55 (1.01–2.37)^‡^		1.91 (1.31–2.8)^†^
Overweight		1.37 (1.23–1.53)*		0.88 (0.63–1.23)		1.1 (0.42–2.88)		0.88 (0.35–2.24)		0.97 (0.84–1.12)		1.56 (1.21–2)*		0.98 (0.82–1.18)		0.75 (0.62–0.9)^†^
Obese		2.04 (1.82–2.28)*		1.04 (0.74–1.45)		1.15 (0.43–3.11)		1.62 (0.69–3.81)		1.04 (0.9–1.21)		1.89 (1.47–2.43)*		1.13 (0.94–1.36)		0.75 (0.62–0.91)^†^
**Chromosomal or genetic anomalies**	2.03 (1.17–3.51)^‡^	1.96 (0.79–4.87)	14.43 (7.65–27.23)*	8.69 (2.5–30.27)^†^	28.05 (8.48–92.77)*	53.11 (10.89–258.92)*	165.17 (81.31–335.53)*	221.49 (74.74–656.36)*	9,238,304,805 (0–)	9,600,000,000 (0–)	0.39 (0.05–2.85)	0 (0–)	6.5 (3.72–11.38)*	6.84 (2.81–16.64)*	8.1 (4.66–14.09)*	10.05 (4.15–24.32)*
Maternal age																
20–34 years		Ref		Ref		Ref		(–)		Ref		Ref		Ref		Ref
< 20 years		0.55 (0.36–0.83)^†^		1 (0.36–2.75)		0 (0–)		0 (0–)		0.72 (0.45–1.15)		1.22 (0.53–2.82)		1.01 (0.6–1.72)		1.32 (0.84–2.08)
≥ 35 years		1.6 (1.44–1.78)*		1.33 (0.95–1.86)		1.19 (0.44–3.23)		1.66 (0.68–4.07)		1.35 (1.17–1.55)*		0.96 (0.76–1.22)		1.33 (1.13–1.56)^†^		1.14 (0.95–1.36)
Parity																
Nulliparous		Ref		Ref		Ref		(–)		Ref		Ref		Ref		Ref
Parity ≥ 1		1.08 (0.98–1.2)		0.58 (0.43–0.79)*		0.9 (0.34–2.41)		1.77 (0.5–6.28)		0.59 (0.52–0.67)*		1.62 (1.26–2.08)*		0.83 (0.71–0.97)^‡^		0.65 (0.56–0.76)*
Maternal BMI																
Normal		Ref		Ref		Ref		Ref		Ref		Ref		Ref		Ref
Underweight		0.57 (0.41–0.8)^†^		0.67 (0.24–1.86)		0 (0–)		0.92 (0.09–9.5)		0.96 (0.67–1.39)		0.63 (0.25–1.55)		1.2 (0.8–1.82)		1.48 (1.02–2.13)^‡^
Overweight		1.32 (1.18–1.47)*		0.99 (0.7–1.4)		0.98 (0.36–2.68)		0.57 (0.18–1.77)		1.06 (0.92–1.22)		1.47 (1.14–1.89)^†^		1.08 (0.91–1.28)		0.92 (0.77–1.08)
Obese		1.9 (1.71–2.12)*		1.26 (0.89–1.77)		0.94 (0.33–2.69)		1.2 (0.45–3.24)		1.24 (1.07–1.43)^†^		1.77 (1.37–2.27)*		1.36 (1.15–1.61)*		1.06 (0.89–1.26)
**CNS anomalies**	1.95 (0.81–4.68)	1.48 (0.58–3.79)	28.12 (11.45–69.05)*	23.37 (9.15–59.72)*	112.21 (36.35–346.37)*	81.28 (24.45–270.19)*	178.93 (62.45–512.69)*	130.82 (40.13–426.43)*	9,238,304,805 (0–)	8,000,000,000 (0–)	0 (0–)	0 (0–)	9.29 (3.86–22.35)*	8.09 (3.04–21.53)*	10.96 (4.45–27.01)*	11.03 (4.18–29.15)*
Pregnancy type																
Singleton		Ref		Ref		Ref		(–)		Ref		Ref		Ref		Ref
Multiple		8.96 (7.64–10.51)*		4.37 (3.44–5.55)*		6.04 (3.19–11.44)*		10.91 (5.94–20.04)*		6.78 (5.96–7.72)*		0.02 (0–0.13)*		25.64 (22.28–29.5)*		26.84 (23.32–30.88)*
**CVS anomalies**	2.61 (1.81–3.77)*	2.28 (1.56–3.35)*	7.17 (4.26–12.07)*	6.16 (3.6–10.53)*	15.89 (5.66–44.58)*	12.85 (4.47–36.98)*	34.16 (15.09–77.32)*	25.74 (11–60.24)*	50.04 (27.51–91.02)*	49.39 (27.05–90.21)*	0.88 (0.36–2.15)	0.98 (0.4–2.42)	4.83 (3.29–7.09)*	4.47 (2.9–6.89)*	5.73 (3.93–8.37)*	5.56 (3.64–8.48)*
Pregnancy type																
Singleton		Ref		Ref		Ref		(–)		Ref		Ref		Ref		Ref
Multiple		6.94 (5.87–8.22)*		3.81 (2.85–5.09)*		7.22 (3.5–14.91)*		7.5 (3.53–15.92)*		5.6 (4.83–6.49)*		0.02 (0–0.15)*		19.75 (16.88–23.1)*		20.76 (17.74–24.3)*
Pregnancy mode																
Spontaneous		Ref		Ref		Ref		(–)		Ref		Ref		Ref		Ref
Assisted conception		2.02 (1.72–2.37)*		1.26 (0.89–1.78)		0.49 (0.15–1.55)		0.93 (0.35–2.47)		1.56 (1.31–1.85)*		0.81 (0.52–1.27)		1.91 (1.58–2.32)*		1.92 (1.58–2.33)*
**Facial anomalies**	0.78 (0.24–2.48)		3.24 (0.42–24.84)		0 (0–)		0 (0–)		14.3 (4.48–45.61)*		3.28 (0.73–14.66)		3.72 (1.16–11.86)^‡^		1.64 (0.37–7.35)	
**Renal anomalies**	0.87 (0.44–1.71)	1.02 (0.32–3.28)	0 (0–)	0 (0–)	0 (0–)	0 (0–)	0 (0–)	0 (0–)	2.25 (1.12–4.52)^‡^	1.9 (0.59–6.1)	0 (0–)	0 (0–)	1.37 (0.53–3.5)	1.34 (0.3–6.02)	1.13 (0.4–3.18)	1.07 (0.24–4.86)
Nationality																
Qatari		1 (0.89–1.13)		1.59 (1.11–2.29)^‡^		1.22 (0.36–4.1)		4.33 (1.14–16.42)^‡^		1.19 (1.02–1.39)^‡^		0.75 (0.55–1.02)		1.75 (1.47–2.09)*		2.18 (1.83–2.6)*
Other arabs		0.94 (0.85–1.03)		1.04 (0.74–1.45)		1.16 (0.42–3.24)		1.57 (0.4–6.15)		0.97 (0.85–1.1)		1.52 (1.21–1.9)*		1.05 (0.89–1.23)		0.91 (0.77–1.08)
Other nationalities		Ref		Ref		Ref		Ref		Ref		Ref		Ref		Ref
Parity																
Nulliparous		Ref		Ref		Ref		Ref		Ref		Ref		Ref		Ref
Parity ≥ 1		1.19 (1.08–1.31)		0.6 (0.45–0.8)^†^		0.95 (0.36–2.51)		2.04 (0.58–7.22)		0.62 (0.55–0.7)*		1.59 (1.24–2.04)*		0.85 (0.73–0.99)^‡^		0.65 (0.56–0.75)*
Diabetes																
None		Ref		Ref		Ref		(–)		Ref		Ref		Ref		Ref
GDM		0.98 (0.89–1.07)		1 (0.74–1.35)		1 (0.39–2.54)		1.09 (0.42–2.84)		1.08 (0.95–1.22)		1.05 (0.86–1.29)		1 (0.87–1.16)		0.86 (0.74–1)
PGDM		2.72 (2.16–3.43)*		2.31 (1.36–3.93)^†^		1.36 (0.17–10.7)		0 (0–)		2.41 (1.88–3.09)*		1.21 (0.75–1.95)		2.38 (1.81–3.13)*		1.24 (0.88–1.73)
Maternal BMI																
Normal		Ref		Ref		Ref		Ref		Ref		Ref		Ref		Ref
Underweight		0.55 (0.39–0.76)*		0.7 (0.25–1.94)		0 (0–)		2.42 (0.29–20.3)		0.95 (0.66–1.36)		0.65 (0.26–1.61)		1.19 (0.78–1.81)		1.46 (1–2.11)^‡^
Overweight		1.35 (1.21–1.5)*		1 (0.71–1.41)		1.17 (0.42–3.28)		0.58 (0.16–2.1)		1.05 (0.92–1.21)		1.45 (1.13–1.87)^†^		1.06 (0.89–1.25)		0.89 (0.75–1.05)
Obese		1.97 (1.77–2.21)*		1.16 (0.81–1.65)		0.93 (0.29–2.94)		1.11 (0.36–3.35)		1.19 (1.03–1.38)^‡^		1.71 (1.32–2.21)*		1.25 (1.05–1.49)^‡^		0.98 (0.82–1.17)
Other congenital anomalies	1.11 (0.47–2.65)	0.66 (0.25–1.77)	4.22 (0.98–18.09)	2.7 (0.6–12.08)	44.88 (10.24–196.67)*	28.88 (6–139.08)*	25.56 (3.37–194.02)^†^	13.85 (1.64–116.92)^‡^	57.19 (13.36–244.76)*	49.89 (11.47–216.98)*	0.94 (0.13–6.97)	0.97 (0.13–7.32)	3.48 (1.36–8.91)	1.74 (0.52–5.82)	3.7 (1.45–9.47)^†^	2.15 (0.64–7.2)
Nationality																
Qatari		0.97 (0.9–1.04)		1.01 (0.8–1.26)		0.87 (0.42–1.83)		1.87 (0.89–3.93)		0.9 (0.82–0.99)^‡^		0.81 (0.68–0.97)^‡^		1.15 (1.02–1.29)^‡^		1.21 (1.08–1.37)^†^
Other arabs		0.97 (0.91–1.03)		0.95 (0.78–1.15)		1 (0.54–1.84)		0.94 (0.44–2)		0.88 (0.81–0.95)^†^		1.45 (1.27–1.65)*		0.79 (0.71–0.88)*		0.65 (0.58–0.73)*
Other nationalities		Ref		Ref		Ref		Ref		Ref		Ref		Ref		Ref
Pregnancy type																
Singleton		Ref		Ref		Ref		(–)		Ref		Ref		Ref		Ref
Multiple		7.08 (5.98–8.38)*		3.92 (2.93–5.26)*		7.98 (3.83–16.61)*		13.73 (6.82–27.64)*		5.78 (4.98–6.7)*		0.02 (0–0.15)*		20.26 (17.3–23.74)*		21.71 (18.5–25.48)*
Pregnancy mode																
Spontaneous		Ref		Ref		Ref		(–)		Ref		Ref		Ref		Ref
Assisted conception		2.02 (1.72–2.38)*		1.25 (0.88–1.79)		0.49 (0.15–1.59)		0.47 (0.16–1.37)		1.55 (1.31–1.84)*		0.81 (0.51–1.3)		1.85 (1.52–2.24)*		1.85 (1.52–2.26)*
Gender																
Male		1.09 (1.03–1.15)		1.23 (1.04–1.45)^‡^		1.23 (0.72–2.1)		1.26 (0.7–2.27)		1.25 (1.17–1.35)*		1.66 (1.47–1.88)*		1.17 (1.06–1.28)^†^		0.82 (0.75–0.91)*
Female		Ref		Ref		Ref		(–)		Ref		Ref		Ref		Ref
**Multiple congenital anomalies**	2 (1.25–3.22)	0.96 (0.4–2.31)	18.45 (10.98–31.02)*	13 (4.96–34.09)*	76.07 (37.01–156.38)*	38.34 (7.91–185.8)*	324.58 (184.23–571.85)*	250.12 (84.89–736.89)*	9,238,304,805 (0–)	7,780,000,000 (0–)	0 (0–)	0 (0–)	7.15 (4.43–11.53)*	4.12 (1.66–10.23)^†^	10.76 (6.7–17.3)*	7.31 (3.04–17.6)*
Parity																
Nulliparous		Ref		Ref		Ref		(–)		Ref		Ref		Ref		Ref
Parity ≥ 1		1.27 (1.15–1.41)*		0.62 (0.47–0.83)^†^		0.78 (0.32–1.92)		1.59 (0.58–4.38)		0.67 (0.59–0.75)*		1.52 (1.19–1.95)^†^		1.02 (0.87–1.18)		0.78 (0.67–0.91)^†^
Diabetes																
None		Ref		Ref		Ref		(–)		Ref		Ref		Ref		Ref
GDM		0.98 (0.9–1.08)		0.97 (0.72–1.3)		0.91 (0.36–2.26)		1.14 (0.48–2.73)		1.07 (0.95–1.21)		1.05 (0.86–1.29)		0.98 (0.84–1.14)		0.82 (0.7–0.96)^‡^
PGDM		2.83 (2.24–3.56)*		2.24 (1.32–3.82)^†^		1.02 (0.13–8.14)		0 (0–)		2.52 (1.96–3.23)*		1.09 (0.68–1.76)		2.67 (2.02–3.53)*		1.36 (0.96–1.92)
Pregnancy mode																
Spontaneous		Ref		Ref		Ref		(–)		Ref		Ref		Ref		Ref
Assisted conception		3.43 (2.83–4.17)*		1.64 (1.01–2.65)^‡^		2.27 (0.63–8.16)		1.52 (0.36–6.47)		3.11 (2.55–3.79)*		0.21 (0.08–0.5)*		7.67 (6.29–9.35)*		8.17 (6.7–9.97)*
Maternal BMI																
Normal		Ref		Ref		Ref		(–)		Ref		Ref		Ref		Ref
Underweight		0.55 (0.39–0.77)*		0.67 (0.24–1.86)		0 (0–)		1.22 (0.12–11.91)		0.99 (0.68–1.42)		0.63 (0.25–1.55)		1.27 (0.83–1.95)		1.57 (1.08–2.29)^‡^
Overweight		1.33 (1.2–1.48)*		1.01 (0.72–1.41)		1.35 (0.49–3.68)		0.69 (0.23–2.07)		1.04 (0.91–1.2)		1.47 (1.15–1.9)^†^		1.05 (0.88–1.24)		0.86 (0.73–1.03)
Obese		1.89 (1.69–2.11)*		1.17 (0.82–1.65)		1.03 (0.34–3.13)		1.09 (0.39–3)		1.15 (1–1.33)		1.77 (1.37–2.28)*		1.2 (1.01–1.43)^‡^		0.93 (0.77–1.11)

*CNS* central nervous system; *CVS* cardiovascular system; others: gastrointestinal, respiratory, urogenital, and skeletal; multiple: two or more anomalies; *GDM* gestational diabetes mellitus; *PGDM* pregestational diabetes mellitus; *NA* not applicable.

*p < 0.001; ^†^p < 0.01; ^‡^p < 0.05.

#### Chromosomal/genetic anomalies

Chromosomal/genetic anomalies were found to be significantly associated with Apgar < 7 at 1 min and at 5 min, in hospital mortality, prematurity, and low birth weight before and after adjusting for maternal age, parity, and maternal BMI. In addition, chromosomal/genetic anomalies were significantly associated with caesarean delivery only before the adjustment of the other factors (Table 3).

#### Central nervous system (CNS) anomalies

CNS anomalies were found to be significantly associated with Apgar < 7 at 1 min and at 5 min, in hospital mortality, prematurity and low birth weight, before and after adjusting for pregnancy mode (Table 3).

#### Cardiovascular system (CVS) anomalies

CVS anomalies were found to be significantly associated with higher rate of caesarean deliveries, Apgar < 7 at 1 min and at 5 min, in hospital mortality, admission to NICU or perinatal death in LR/OT, prematurity and low birth weight, before and after adjusting for pregnancy type and pregnancy mode (Table 3).

#### Congenital facial anomalies

Facial anomalies were found to be significantly associated with admission to NICU or perinatal death in LR/OT and prematurity (Table 3). The multiple logistic regression analysis was not performed as there were no significant risk factors in the first stage.

#### Congenital renal anomalies

Renal anomalies were found to be significantly associated with admission to NICU or perinatal death in LR/OT in the univariate analysis. However, after adjusting for nationality, parity, diabetic status, and maternal BMI at birth the relationship became non-significant (Table 3).

#### Other congenital anomalies (GIT, respiratory, urogenital, and skeletal anomalies)

GIT, respiratory, urogenital, and skeletal anomalies were found to be significantly associated with Apgar < 7 at 5 min, in, hospital mortality, and admission to NICU or perinatal death in LR/OT, before and after adjusting for nationality, pregnancy type, pregnancy mode, and baby gender. In addition, other anomalies were significantly associated with low birth weight only before the adjustment of the other factors (Table 3).

#### Multiple congenital anomalies (MCAs)

MCAs were found to be significantly associated with Apgar < 7 at 1 min and at 5 min, in hospital mortality, prematurity and low birth weight before and after adjustment for parity, diabetes, pregnancy mode, and maternal BMI (Table 3).

## Discussion

This large population-based study is the first of its kind to assess the prevalence, maternal risk factors and neonatal outcomes associated with CAs in Qatar.

In the 12 months from April 2017, we estimate the prevalence of CAs was 130/10,000 live births. This is similar to that reported in European countries^25^, and is slightly lower than that reported by the European Surveillance of Congenital Anomalies registry (199.85 per 10,000 live births)^26^, British Isles Network of Congenital Anomaly Registers (BINOCAR) (206 per 10,000 births)^27^. In the March of Dimes Foundation report in 2006, Qatar ranked 16th globally for the number of CA cases per 1000 live births (~ 73.4 per 1000 live births)^5^. The prevalence of CAs was estimated to be 20 per 1000 live births in Egypt^19^, and 412 per 10,000 births in Saudi Arabia^18^. In our cohort, in-hospital mortality associated with CAs was 15.4% (Table 1), and, during 2018 accounted for 2.1% of total deaths^9^. This value is much lower compared to that reported from Egypt which was reported to be 15% of all infant deaths^19^. A study conducted in Saudi Arabia estimated the mortality rate at the age of 2 years to be 15.8%^18^. This variation in death rates associated with CAs may be explained by social, ecological, and economical influences^6,7^. In addition, racial and ethnic disparities were reported to largely contribute to increased CA-related infant mortalities^28,29^.

The most common CAs were CVS (n = 117; 35%), MCAs (n = 69, 21%), chromosomal/genetic (51; 15%), renal (n = 39; 12%), CNS (n = 20; 6%), and facial (14, 4%) anomalies (Appendix 2). These findings are in concordance with March of Dimes Foundation report in 2006, which showed that CVS anomalies, particularly congenital heart diseases (CHDs), are the most common severe birth defects around the globe^5^.

The multivariable regression analyses revealed that nationality, maternal age, multiple pregnancy, parity, assisted conception as pregnancy mode, and underweight and obesity were significantly associated with at least one type of CA. These are all well-established risk factors for several major CAs among different racial and ethnic groups^11,15–19,30,31^.

In our study cohort, CAs were independently associated with high rates of caesarean deliveries, poorer condition at birth, prematurity, low birthweight, increased in-hospital mortality, and more need specialist care in the delivery room or in NICU. Previous studies showed that CAs were more likely to be risk factors of premature birth, specifically extremely, moderate and late preterm birth in comparison to full term birth. CAs were also more likely to be risk factors for babies born small for gestational age in comparison to appropriate for gestational age before the adjustment for other risk factors^23^. In our study on the other hand, we were able to find which specific CAs were related to prematurity and birth weight. Reports from high-income countries estimate that up to 70% of CAs can either be prevented, or that affected infants can be offered care that could be lifesaving or that would reduce the severity of disability^5^. Our findings indicate that there is a great need for a specialized congenital anomaly data registry to identify risk factors and outcomes. Furthermore, counselling of mothers and their families during pre-conception when considering subsequent pregnancies and during antenatal or postnatal may help to identify specific needs for pregnant women and their babies.

Overall risk of having a child with a CA could be reduced with planning and preparation for pregnancy, particularly in higher risk groups. Therefore, many potential pre-pregnancy interventions, such as supplementations, review of current medications, along with diet and physical activity advice during preparation for next pregnancy, are likely to reduce the risk of CAs. For example, the US reported a remarkable 46% decline in infant mortality rates from birth defects over the period 1980 to 2001 where much of this reduction can be attributed to improvements in diagnosis, care and prevention. Other high-income countries have reported similar declines^5^. Therefore, there is a need for a more accurate assessment of risk factors and complications that may help to identify the appropriate needs and interventions. As indicated in our study, Cardiovascular System (CVS) were the most common CAs. Precision medicine presents an opportunity to improve clinical outcomes through a unified diagnostics path, genetic testing utility, genetic counseling, and personalized management. For instance, in the case of CHDs, precision medicine narrows existing gaps between the laboratory bench and the clinic by dissecting molecular mechanisms^32^.

Our study has several strengths. This study used data from the PEARL-Peristat Study (Perinatal Neonatal Registry)^22,23^. This registry reports data on maternal, neonatal and perinatal mortality, morbidities, and their correlates, including data on live births and neonatal mortality from all public and private maternity facilities in Qatar^21–23^. This database is large enough to allow us to study major and minor CAs, with a sample size that is generally representative of births in Qatar. In addition, HMC is the main provider of secondary and tertiary healthcare in Qatar, comprising the main national hospital and multiple regional hospitals that are spread across Qatar, and provide care for the majority of births in the country. Furthermore, selection bias was minimized via examining all available live births for the study period.

Despite being the largest study of its kind in the State of Qatar, this study has some limitations. Firstly, stillbirths were excluded due to incomplete data, as there was no certainty of the presence or absence of CAs. Perinatal autopsy and postpartum x-ray or MRI imaging are not routinely performed in Qatar, and therefore the actual cause of a stillbirth can often be unconfirmed. This is a particular concern with an un-booked patients, which is a common problem.

Another factor to consider is the small number of major congenital abnormalities, particularly in the expat community, who will undergo termination of pregnancy abroad and therefore will not be included in this registry of livebirths.

Secondly, the absolute numbers of some of the selected anomalies were low and other anomalies could not be explored. Similarly, the share of the missing data for consanguinity and maternal BMI is very high and therefore, some results need to be treated with caution. Moreover, as with all observational studies, we cannot ascertain causal relationships, but only identify associations. The PEARL-Peristat study was designed to capture a wide range of maternal and neonatal outcomes and was not primarily designed as a CA registry. Potential issues arise from using routine health data include misclassification bias, missing data from incomplete and variable documentation including measurement bias. Finally, other factors (e.g., smoking, alcohol, substance abuse, medication) that can affect the relationship between the risk factors and CA were not included in the study. The reliability of these factors is not high and were not recorded properly for the current sample, hence they were not included in the analysis.

## Conclusion

This large population-based study is the first to assess the prevalence, risk factors and outcomes associated with CAs in the state of Qatar. Multi-factor interactions of demographic and medical factors, including multiple pregnancies were found to be significantly associated with CAs. In addition, CAs were significantly associated with more caesarean deliveries, low Apgar score, increased risk of in-hospital mortality and admission to NICU and perinatal death in labor room and operation theatre, prematurity, and low birth weight. In combination with diagnostic imaging and biochemical parameters of disease progression, the findings of this study may serve as a basis to help make better clinical decisions with accurate assessment of risk factors, complications, and realistic predictions of CAs. There is a need for a specialized congenital anomaly data registry to identify risk factors and outcomes, and the need to improve the counselling services for mothers and their families to identify specific needs for pregnant women and their babies.

## Supplementary Information

Supplementary Information 1.

Supplementary Information 2.

## Data Availability

This is a research article and all data generated or analyzed during this study are included in this published article [and its supplementary information files]. All enquiries should be directed to Nader Al-Dewik: naldewik@hamad.qa.

## Abbreviations

CAs: Congenital anomalies
WHO: World Health Organization
PEARL study: Perinatal neonatal outcomes research study in the Arabian Gulf
CHD: Congenital heart defect
CNS: Central nervous system
OR: Odds ratio
CI: Confidence interval
DM: Diabetes mellitus
PGDM: Pre-gestational diabetes mellitus
