# Supplementary material for: Prevalence, predictors, and outcomes of major congenital anomalies: A population-based register study
Source: Sci Rep. 2023 Feb 7;13:2198. doi: 10.1038/s41598-023-27935-3 (PMC9905082; doi:10.1038/s41598-023-27935-3)
Supplement: Supplementary file 1 — Supplementary Information 1. [file 41598_2023_27935_MOESM1_ESM.docx]

# Appendix 1: Congenital anomalies (CAs) by system according to ICD-10 classification.

**ICD 10**

**“Chromosomal/Genetic” (n=51)**

***Chromosomal abnormalities not elsewhere classified***

Q90 Down syndrome

Q91 Trisomy 18 and Trisomy 13

Q92 Other trisomies and partial trisomies of the autosomes, not elsewhere classified

Q93 Monosomies and deletions from the autosomes, not elsewhere classified

Q95 Balanced rearrangements and structural markers, not elsewhere classified

Q96 Turner's syndrome

Q97 Other sex chromosome abnormalities, female phenotype, not elsewhere classified

Q98 Other sex chromosome abnormalities, male phenotype, not elsewhere classified

Q99 Other chromosome abnormalities, not elsewhere classified

**“Central Nervous System (CNS)” (n=20)**

## *Congenital malformations of the nervous system Q00-Q07*

Q00 Anencephaly and similar malformations

Q01 Encephalocele

Q02 Microcephaly

Q03 Congenital hydrocephalus ✓

Q04 Other congenital malformations of brain

Q05 Spina bifida

Q06 Other congenital malformations of spinal cord

Q07 Other congenital malformations of nervous system

## “Cardiovascular System (CVS)” (n=117)

## *Congenital malformations of the circulatory system Q20-Q28*

***Congenital malformations of cardiac chambers***

Q20.0 Common arterial trunk

Q20.1 Double outlet right ventricle

Q20.2 Double outlet left ventricle

Q20.3 Discordant ventriculoarterial connection

Q20.4 Double inlet ventricle

Q20.5 Discordant atrioventricular connection

Q20.6 Isomerism of atrial appendages

Q20.8 Other congenital malformations of cardiac chambers and connections

Q20.9 Congenital malformation of cardiac chambers and connections, unspecified

***Q21 Congenital malformations of cardiac septa***

Q21.0 Ventricular septal defect

Q21.1 Atrial septal defect

Q21.2 Atrioventricular septal defect

Q21.3 Tetralogy of Fallot

Q21.4 Aortopulmonary septal defect

Q21.8 Other congenital malformations of cardiac septa

Q21.9 Congenital malformation of cardiac septum, unspecified

***Q22 Congenital malformations of pulmonary and tricuspid valves***

Q22.0 Pulmonary valve atresia

Q22.1 Congenital pulmonary valve stenosis

Q22.2 Congenital pulmonary valve insufficiency

Q22.3 Other congenital malformations of pulmonary valve

Q22.4 Congenital tricuspid stenosis

Q22.5 Ebstein's anomaly

Q22.6 Hypoplastic right heart syndrome

Q22.8 Other congenital malformations of tricuspid valve

Q22.9 Congenital malformation of tricuspid valve, unspecified

***Q22 Congenital malformations of pulmonary and tricuspid valves***

Q22.0 Pulmonary valve atresia

Q22.1 Congenital pulmonary valve stenosis

Q22.2 Congenital pulmonary valve insufficiency

Q22.3 Other congenital malformations of pulmonary valve

Q22.4 Congenital tricuspid stenosis

Q22.5 Ebstein's anomaly

Q22.6 Hypoplastic right heart syndrome

Q22.8 Other congenital malformations of tricuspid valve

Q22.9 Congenital malformation of tricuspid valve, unspecified

***Q23 Congenital malformations of aortic and mitral valves***

Q23.0 Congenital stenosis of aortic valve

Q23.1 Congenital insufficiency of aortic valve

Q23.2 Congenital mitral stenosis

Q23.3 Congenital mitral insufficiency

Q23.4 Hypoplastic left heart syndrome

Q23.8 Other congenital malformations of aortic and mitral valves

Q23.9 Congenital malformation of aortic and mitral valves, unspecified

***Other congenital malformations of heart***

Type 1 Excludes

endocardial fibroelastosis (I42.4)

***Q24 Other congenital malformations of heart***

Q24.0 Dextrocardia

Q24.1 Levocardia

Q24.2 Cor triatriatum

Q24.3 Pulmonary infundibular stenosis

Q24.4 Congenital subaortic stenosis

Q24.5 Malformation of coronary vessels

Q24.6 Congenital heart block

Q24.8 Other specified congenital malformations of heart

Q24.9 Congenital malformation of heart, unspecified

***Q25 Congenital malformations of great arteries***

Q25.0 Patent ductus arteriosus

Q25.1 Coarctation of aorta

Q25.2 Atresia of aorta

Q25.21 Interruption of aortic arch

Q25.29 Other atresia of aorta

Q25.3 Supravalvular aortic stenosis

Q25.4 Other congenital malformations of aorta

Q25.40 Congenital malformation of aorta unspecified

Q25.41 Absence and aplasia of aorta

Q25.42 Hypoplasia of aorta

Q25.43 Congenital aneurysm of aorta

Q25.44 Congenital dilation of aorta

Q25.45 Double aortic arch

Q25.46 Tortuous aortic arch

Q25.47 Right aortic arch

Q25.48 Anomalous origin of subclavian artery

Q25.49 Other congenital malformations of aorta

Q25.5 Atresia of pulmonary artery

Q25.6 Stenosis of pulmonary artery

Q25.7 Other congenital malformations of pulmonary artery

Q25.71 Coarctation of pulmonary artery

Q25.72 Congenital pulmonary arteriovenous malformation

Q25.79 Other congenital malformations of pulmonary artery

Q25.8 Other congenital malformations of other great arteries

Q25.9 Congenital malformation of great arteries, unspecified

***Q26 Congenital malformations of great veins***

Q26.0 Congenital stenosis of vena cava

Q26.1 Persistent left superior vena cava

Q26.2 Total anomalous pulmonary venous connection

Q26.3 Partial anomalous pulmonary venous connection

Q26.4 Anomalous pulmonary venous connection, unspecified

Q26.5 Anomalous portal venous connection

Q26.6 Portal vein-hepatic artery fistula

Q26.8 Other congenital malformations of great veins

Q26.9 Congenital malformation of great vein, unspecified

***Q27 Other congenital malformations of peripheral vascular system***

Q27.0 Congenital absence and hypoplasia of umbilical artery

Q27.1 Congenital renal artery stenosis

Q27.2 Other congenital malformations of renal artery

Q27.3 Arteriovenous malformation (peripheral)

Q27.30 Arteriovenous malformation, site unspecified

Q27.31 Arteriovenous malformation of vessel of upper limb

Q27.32 Arteriovenous malformation of vessel of lower limb

Q27.33 Arteriovenous malformation of digestive system vessel

Q27.34 Arteriovenous malformation of renal vessel

Q27.39 Arteriovenous malformation, other site

Q27.4 Congenital phlebectasia

Q27.8 Other specified congenital malformations of peripheral vascular system

Q27.9 Congenital malformation of peripheral vascular system, unspecified

***Q28 Other congenital malformations of circulatory system***

Q28.0 Arteriovenous malformation of precerebral vessels

Q28.1 Other malformations of precerebral vessels

Q28.2 Arteriovenous malformation of cerebral vessels

Q28.3 Other malformations of cerebral vessels

Q28.8 Other specified congenital malformations of circulatory system

Q28.9 Congenital malformation

**“Facial” (n=14)**

Cleft lip and cleft palate

Q35 Cleft palate

Q36 Cleft lip

Q37 Cleft palate with cleft lip

**“Renal” (n=39)**

***Congenital malformations of the urinary system***

Q60 Renal agenesis and other reduction defects of kidney

Q61 Cystic kidney disease

Q62 Congenital obstructive defects of renal pelvis and congenital malformations of ureter

Q63 Other congenital malformations of kidney

Q64 Other congenital malformations of urinary system

**“Others” (n=22)**

***Other congenital malformations of the digestive system***

Q38 Other congenital malformations of tongue, mouth and pharynx

Q39 Congenital malformations of esophagus

Q40 Other congenital malformations of upper alimentary tract

Q41 Congenital absence, atresia and stenosis of small intestine

Q42 Congenital absence, atresia and stenosis of large intestine

Q43 Other congenital malformations of intestine

Q44 Congenital malformations of gallbladder, bile ducts and liver

Q45 Other congenital malformations of digestive system

***Congenital malformations of the respiratory system***

Q30 Congenital malformations of nose

Q31 Congenital malformations of larynx

Q32 Congenital malformations of trachea and bronchus

Q33 Congenital malformations of lung

Q34 Other congenital malformations of respiratory system

***Congenital malformations of genital organs***

Q50 Congenital malformations of ovaries, fallopian tubes and broad ligaments

Q51 Congenital malformations of uterus and cervix

Q52 Other congenital malformations of female genitalia

Q53 Undescended and ectopic testicle

Q54 Hypospadias

Q55 Other congenital malformations of male genital organs

Q56 Indeterminate sex and pseudohermaphroditism

***Congenital malformations and deformations of the musculoskeletal system***

Q65 Congenital deformities of hip

Q66 Congenital deformities of feet

Q67 Congenital musculoskeletal deformities of head, face, spine and chest

Q68 Other congenital musculoskeletal deformities

Q69 Polydactyly

Q70 Syndactyly

Q71 Reduction defects of upper limb

Q72 Reduction defects of lower limb

Q73 Reduction defects of unspecified limb

Q74 Other congenital malformations of limb(s)

Q75 Other congenital malformations of skull and face bones

Q76 Congenital malformations of spine and bony thorax

Q77 Osteochondrodysplasia with defects of growth of tubular bones and spine

Q78 Other osteochondrodysplasias

Q79 Congenital malformations of musculoskeletal system, not elsewhere classified

**“Multiple” (n=69)**

Carrying more than one type of congenital anomaly (from those indicated above).
