# Supplementary material for: Prevalence, predictors, and outcomes of major congenital anomalies: A population-based register study
Source: Sci Rep. 2023 Feb 7;13:2198. doi: 10.1038/s41598-023-27935-3 (PMC9905082; doi:10.1038/s41598-023-27935-3)
Supplement: Supplementary file 2 — Supplementary Information 2. [file 41598_2023_27935_MOESM2_ESM.docx]

| **Risk factors and outcomes** | Total (n=25,073) | Chromosomal/  genetics (n=51) | CNS (n=20) | | CVS (n=117) | Facial (n=14) | GIT (n=7) | Renal (n=39) | Respiratory (n=7) | Urogenital (n=2) | Skeletal (n=6) | MCAs (n=69) | None (n=24741) |
| --- | --- | --- | --- | --- | --- | --- | --- | --- | --- | --- | --- | --- | --- |
|  | n (%) | n(%) | n (%) | n (%) | | n (%) | n (%) | n (%) | n (%) | n (%) | n (%) | n (%) | n (%) |
| **Nationality†** |  |  |  |  | |  |  |  |  |  |  |  |  |
| Qatari | 5863 (23.4) | 11 (21.6) | 3 (15) | 34 (29.1) | | 3 (21.4) | 1 (14.3) | 14 (35.9) | 3 (42.9) | 0 (0) | 1 (16.7) | 23 (33.3) | 5770 (23.3) |
| Other Arabs | 10621 (42.4) | 19 (37.3) | 12 (60) | 45 (38.5) | | 8 (57.1) | 6 (85.7) | 17 (43.6) | 3 (42.9) | 2 (100) | 4 (66.7) | 22 (31.9) | 10483 (42.4) |
| Other Nationalities | 8585 (34.2) | 21 (41.2) | 5 (25) | 38 (32.5) | | 3 (21.4) | 0 (0) | 8 (20.5) | 1 (14.3) | 0 (0) | 1 (16.7) | 24 (34.8) | 8484 (34.3) |
|  |  |  |  |  | |  |  |  |  |  |  |  |  |
| **Maternal age** |  |  |  |  | |  |  |  |  |  |  |  |  |
| 20-34 years | 19829 (79.1) | 29 (56.9) | 18 (90) | 91 (77.8) | | 12 (85.7) | 5 (71.4) | 30 (76.9) | 7 (100) | 1 (50) | 5 (83.3) | 52 (75.4) | 19579 (79.1) |
| <20 years | 488 (1.9) | 0 (0) | 0 (0) | 1 (0.9) | | 1 (7.1) | 0 (0) | 1 (2.6) | 0 (0) | 0 (0) | 0 (0) | 3 (4.3) | 482 (1.9) |
| ≥35 years | 4756 (19) | 22 (43.1) | 2 (10) | 25 (21.4) | | 1 (7.1) | 2 (28.6) | 8 (20.5) | 0 (0) | 1 (50) | 1 (16.7) | 14 (20.3) | 4680 (18.9) |
|  |  |  |  |  | |  |  |  |  |  |  |  |  |
| **Pregnancy type** |  |  |  |  | |  |  |  |  |  |  |  |  |
| Singleton | 24036 (95.9) | 51 (100) | 16 (80) | 102 (87.2) | | 13 (92.9) | 5 (71.4) | 35 (89.7) | 5 (71.4) | 1 (50) | 6 (100) | 63 (91.3) | 23739 (96) |
| Multiple | 1037 (4.1) | 0 (0) | 4 (20) | 15 (12.8) | | 1 (7.1) | 2 (28.6) | 4 (10.3) | 2 (28.6) | 1 (50) | 0 (0) | 6 (8.7) | 1002 (4) |
|  |  |  |  |  | |  |  |  |  |  |  |  |  |
| **Parity †** |  |  |  |  | |  |  |  |  |  |  |  |  |
| Nulliparous | 7126 (28.4) | 8 (15.7) | 7 (35) | 30 (25.6) | | 4 (28.6) | 0 (0) | 18 (46.2) | 2 (28.6) | 1 (50) | 1 (16.7) | 29 (42) | 7026 (28.4) |
| Parity ≥1 | 17943 (71.6) | 43 (84.3) | 13 (65) | 87 (74.4) | | 10 (71.4) | 7 (100) | 21 (53.8) | 5 (71.4) | 1 (50) | 5 (83.3) | 40 (58) | 17711 (71.6) |
|  |  |  |  |  | |  |  |  |  |  |  |  |  |
| **Diabetes** |  |  |  |  | |  |  |  |  |  |  |  |  |
| None | 17404 (69.4) | 34 (66.7) | 14 (70) | 79 (67.5) | | 11 (78.6) | 7 (100) | 29 (74.4) | 3 (42.9) | 1 (50) | 5 (83.3) | 51 (73.9) | 17170 (69.4) |
| GDM | 7083 (28.2) | 16 (31.4) | 6 (30) | 33 (28.2) | | 3 (21.4) | 0 (0) | 6 (15.4) | 4 (57.1) | 1 (50) | 1 (16.7) | 13 (18.8) | 7000 (28.3) |
| PGDM | 586 (2.3) | 1 (2) | 0 (0) | 5 (4.3) | | 0 (0) | 0 (0) | 4 (10.3) | 0 (0) | 0 (0) | 0 (0) | 5 (7.2) | 571 (2.3) |
|  |  |  |  |  | |  |  |  |  |  |  |  |  |
| **Chronic Hypertension** |  |  |  |  | |  |  |  |  |  |  |  |  |
| No | 24775 (98.8) | 50 (98) | 20 (100) | 116 (99.1) | | 14 (100) | 7 (100) | 38 (97.4) | 7 (100) | 2 (100) | 6 (100) | 68 (98.6) | 24447 (98.8) |
| Yes | 298 (1.2) | 1 (2) | 0 (0) | 1 (0.9) | | 0 (0) | 0 (0) | 1 (2.6) | 0 (0) | 0 (0) | 0 (0) | 1 (1.4) | 294 (1.2) |
|  |  |  |  |  | |  |  |  |  |  |  |  |  |
| **Consanguinity†** |  |  |  |  | |  |  |  |  |  |  |  |  |
| No | 5897 (69.6) | 11 (61.1) | 3 (60) | 24 (66.7) | | 0 (0) | 2 (66.7) | 9 (60) | 1 (0) | 0 (0) | 0 (50) | 15 (68.2) | 5832 (69.7) |
| Yes | 2579 (30.4) | 7 (38.9) | 2 (40) | 12 (33.3) | | 3 (100) | 1 (33.3) | 6 (40) | 1 (0) | 0 (0) | 0 (50) | 7 (31.8) | 2540 (30.3) |
|  |  |  |  |  | |  |  |  |  |  |  |  |  |
| **Pregnancy mode†** |  |  |  |  | |  |  |  |  |  |  |  |  |
| Spontaneous | 23987 (96.2) | 49 (96.1) | 18 (90) | 105 (90.5) | | 12 (85.7) | 6 (85.7) | 37 (94.9) | 5 (71.4) | 1 (50) | 6 (100) | 60 (88.2) | 23688 (96.3) |
| Assisted conception | 946 (3.8) | 2 (3.9) | 2 (10) | 11 (9.5) | | 2 (14.3) | 1 (14.3) | 2 (5.1) | 2 (28.6) | 1 (50) | 0 (0) | 8 (11.8) | 915 (3.7) |
|  |  |  |  |  | |  |  |  |  |  |  |  |  |
| **Delivery mode** |  |  |  |  | |  |  |  |  |  |  |  |  |
| Vaginal | 16517 (65.9) | 25 (49) | 10 (50) | 50 (42.7) | | 10 (71.4) | 4 (57.1) | 27 (69.2) | 5 (71.4) | 1 (50) | 4 (66.7) | 34 (49.3) | 16347 (66.1) |
| Caesarean | 8556 (34.1) | 26 (51) | 10 (50) | 67 (57.3) | | 4 (28.6) | 3 (42.9) | 12 (30.8) | 2 (28.6) | 1 (50) | 2 (33.3) | 35 (50.7) | 8394 (33.9) |
|  |  |  |  |  | |  |  |  |  |  |  |  |  |
| **Gestational age at birth** |  |  |  |  | |  |  |  |  |  |  |  |  |
| 24-31 weeks | 419 (1.7) | 3 (5.9) | 1 (5) | 23 (19.7) | | 0 (0) | 0 (0) | 3 (7.7) | 1 (14.3) | 0 (0) | 0 (0) | 12 (17.4) | 376 (1.5) |
| 32-36 weeks | 2101 (8.4) | 18 (35.3) | 9 (45) | 17 (14.5) | | 4 (28.6) | 3 (42.9) | 2 (5.1) | 1 (14.3) | 1 (50) | 0 (0) | 18 (26.1) | 2028 (8.2) |
| ≥37 weeks | 22553 (89.9) | 30 (58.8) | 10 (50) | 77 (65.8) | | 10 (71.4) | 4 (57.1) | 34 (87.2) | 5 (71.4) | 1 (50) | 6 (100) | 39 (56.5) | 22337 (90.3) |
|  |  |  |  |  | |  |  |  |  |  |  |  |  |
| **Birth weight†** |  |  |  |  | |  |  |  |  |  |  |  |  |
| ≤2499g | 2400 (9.6) | 23 (45.1) | 10 (52.6) | 43 (36.8) | | 2 (14.3) | 2 (28.6) | 4 (10.3) | 2 (28.6) | 2 (100) | 0 (0) | 36 (52.2) | 2276 (9.2) |
| ≥2500g | 22664 (90.4) | 28 (54.9) | 9 (47.4) | 74 (63.2) | | 12 (85.7) | 5 (71.4) | 35 (89.7) | 5 (71.4) | 0 (0) | 6 (100) | 33 (47.8) | 22457 (90.8) |
|  |  |  |  |  | |  |  |  |  |  |  |  |  |
| **Macrosomic Baby†** |  |  |  |  | |  |  |  |  |  |  |  |  |
| <4kg | 23858 (95.2) | 50 (98) | 19 (100) | 112 (95.7) | | 12 (85.7) | 7 (100) | 39 (100) | 7 (100) | 2 (100) | 5 (83.3) | 69 (100) | 23536 (95.2) |
| ≥4kg | 1206 (4.8) | 1 (2) | 0 (0) | 5 (4.3) | | 2 (14.3) | 0 (0) | 0 (0) | 0 (0) | 0 (0) | 1 (16.7) | 0 (0) | 1197 (4.8) |
|  |  |  |  |  | |  |  |  |  |  |  |  |  |
| **Apgar <7 at 1 min** |  |  |  |  | |  |  |  |  |  |  |  |  |
| No | 24438 (97.5) | 38 (74.5) | 12 (60) | 100 (85.5) | | 13 (92.9) | 6 (85.7) | 39 (100) | 6 (85.7) | 2 (100) | 6 (100) | 48 (69.6) | 24168 (97.7) |
| Yes | 635 (2.5) | 13 (25.5) | 8 (40) | 17 (14.5) | | 1 (7.1) | 1 (14.3) | 0 (0) | 1 (14.3) | 0 (0) | 0 (0) | 21 (30.4) | 573 (2.3) |
|  |  |  |  |  | |  |  |  |  |  |  |  |  |
| **Apgar <7 at 5 mins** |  |  |  |  | |  |  |  |  |  |  |  |  |
| No | 24995 (99.7) | 48 (94.1) | 16 (80) | 113 (96.6) | | 14 (100) | 6 (85.7) | 39 (100) | 6 (85.7) | 2 (100) | 6 (100) | 59 (85.5) | 24686 (99.8) |
| Yes | 78 (0.3) | 3 (5.9) | 4 (20) | 4 (3.4) | | 0 (0) | 1 (14.3) | 0 (0) | 1 (14.3) | 0 (0) | 0 (0) | 10 (14.5) | 55 (0.2) |
|  |  |  |  |  | |  |  |  |  |  |  |  |  |
| **Gender†** |  |  |  |  | |  |  |  |  |  |  |  |  |
| Male | 12798 (51.1) | 25 (49) | 7 (35) | 64 (54.7) | | 9 (64.3) | 5 (71.4) | 22 (56.4) | 5 (71.4) | 2 (100) | 5 (83.3) | 35 (50.7) | 12619 (51) |
| Female | 12269 (48.9) | 26 (51) | 13 (65) | 53 (45.3) | | 5 (35.7) | 2 (28.6) | 17 (43.6) | 2 (28.6) | 0 (0) | 1 (16.7) | 34 (49.3) | 12116 (49) |
|  |  |  |  |  | |  |  |  |  |  |  |  |  |
| **Baby outcome†** |  |  |  |  | |  |  |  |  |  |  |  |  |
| Discharged alive | 24974 (99.6) | 39 (76.5) | 15 (75) | 110 (94) | | 14 (100) | 7 (100) | 39 (100) | 6 (85.7) | 2 (100) | 6 (100) | 43 (62.3) | 24693 (99.8) |
| In-hospital Mortality | 97 (0.4) | 12 (23.5) | 5 (25) | 7 (6) | | 0 (0) | 0 (0) | 0 (0) | 1 (14.3) | 0 (0) | 0 (0) | 26 (37.7) | 46 (0.2) |
|  |  |  |  |  | |  |  |  |  |  |  |  |  |
| **Baby disposition†** |  |  |  |  | |  |  |  |  |  |  |  |  |
| Admission to postnatal ward | 21102 (84.2) | 0 (0) | 0 (0) | 12 (10.3) | | 4 (28.6) | 0 (0) | 28 (71.8) | 1 (14.3) | 0 (0) | 1 (16.7) | 0 (0) | 21056 (85.1) |
| Admission to NICU or perinatal death in LR/OT | 3968 (15.8) | 51 (100) | 20 (100) | 105 (89.7) | | 10 (71.4) | 7 (100) | 11 (28.2) | 6 (85.7) | 2 (100) | 5 (83.3) | 69 (100) | 3682 (14.9) |

†Missing data for: nationality (n=4), parity (n=4), consanguinity (n=16597), pregnancy mode (n=140), birth weight/macrosomia (n=9), gender (n=4), baby outcome (n=2). CNS: central nervous system; CHD: congenital heart defects; RESP: respiratory; GIT: gatrointestinal tract; MCAs: multiple congenital anomalies; GDM: gestational diabetes mellitus; PGDM: pregestational diabetes mellitus.
